# International models of accreditation and certification for hospitals with a focus on nursing: a scoping review

**DOI:** 10.1186/s12913-024-11759-6

**Published:** 2024-11-12

**Authors:** Carolin Gurisch, Joan Kleine, Claudia Bettina Maier

**Affiliations:** 1 Department of Healthcare Management, Technische Universität Berlin, Berlin, Germany; 2 Department of Health Sciences, BQS Institut für Qualität und Patientensicherheit GmbH, Hamburg, Germany; 3 School of Public Health, Bielefeld University, Bielefeld, Germany

**Keywords:** Hospitals, Quality of healthcare, Health care quality assurance, Accreditation, Certification, Nursing, Scoping review

## Abstract

**Background:**

Quality assurance in hospitals is essential for ensuring patient safety, quality of care and efficiency. The nursing profession is a key contributor to healthcare quality, yet, a comprehensive overview and comparison of the role and scope of nursing as part of accreditation and certification schemes has been lacking. The aim was to identify if and to what extent international accreditation or certification schemes focus on nursing, and to compare their conceptual models and outcome indicators.

**Methods:**

A scoping review was conducted. A search strategy was developed together with a librarian and carried out in January 2024 in Medline, CINAHL, Web of Science Core Collection, Cochrane Reviews and Google Scholar. Additionally, authoritative websites of accreditation/certification holders were searched. Inclusion criteria were studies on international accreditation or certification schemes for hospital settings with relevance to nursing published in English or German. Screening and data analysis adhered to the Joanna Briggs Institute’s methodology, with reporting following the Preferred Reporting Items for Systematic Reviews and Meta-Analyses extension for Scoping Reviews guideline (PRISMA-ScR).

**Results:**

The search identified 17.315 records. After removing duplicates and screening of titles/abstracts, 336 full-texts remained. A total of 124 studies were included, identifying seven international accreditation/certification schemes: European Foundation for Quality Management, Evaluation and Quality Improvement Program, International Organization for Standardization 9001, Joint Commission International^®^, Magnet Recognition Program^®^, Pathway to Excellence^®^ Program, Qmentum^®^. The different schemes ranged from nursing-specific to having no specific focus on, but relevance for, nursing and varied in their topics, focus on structure, process and outcome quality and structure and content of requirements. Additionally, outcome indicators varied, with differences in the extent to which indicators were nurse-sensitive, compulsoriness of data collection, and use of external benchmarking.

**Conclusions:**

The seven international schemes show large conceptual variations as to their focus on nursing and differences in the degree of nurse-specific outcome indicators. Hospital and nursing managers, policymakers, patients and the public need to understand the content and outcome dimensions of the schemes when making decisions or assessing hospital quality data.

**Supplementary Information:**

The online version contains supplementary material available at 10.1186/s12913-024-11759-6.

## Background

Healthcare quality, as defined by the World Health Organization, encompasses effectiveness, safety and people-centered care [1]. It describes the extent to which intended health outcomes are achieved through health services, including hospital treatment, and overall health system performance [1, 2]. Nurses as the largest occupational group in hospitals play a pivotal role in contributing to healthcare quality [2–5]. Despite their contribution, nursing performance is not always adequately reflected in quality or hospital performance measurements.

There is no universally accepted definition of the components required for the measurement of nursing quality [6]. Nurse-sensitive indicators can be utilized to assess nursing quality (ibid.). While definitions and measures vary, these indicators generally reflect patient outcomes influenced by nursing care [6, 7].

Quality assurance in hospitals is often facilitated by certifications or accreditations [8]. These schemes, either mandatory or voluntary depending on country policies and contexts, comprise some form of assessment against predefined standards by an external organization [9]. Certification usually refers to the assessment of standards of the International Organization for Standardization (ISO), whereas accreditation schemes encompass various forms of standards assessment (ibid.). However, these schemes cannot fully be delineated from one another. There are large differences between and within these schemes regarding accreditation bodies (e.g. national government or autonomous organizations), commercial or governmental financing and methods of (re-)assessment (e.g. conducting audits or using self-reporting) (ibid.).

There is a plethora of accreditation and certification schemes worldwide [10]. A study analyzed the quality and safety of European hospitals by comparing the availability and definition of ten process- and outcome-related indicators across hospitals from five countries [11]. The study identified seven challenges in comparing hospital quality across countries, mostly related to non-uniform definitions and availability of indicators (ibid.). Further challenges relate to the mostly voluntary use of accreditations and certifications in European countries as well as discrepancies in the schemes being utilized (ibid.). Accreditation/certification schemes are either limited to a national context or have an international scope [10]. They may assess overall organizational quality or target specific departments, professions or diseases (ibid.). While some schemes focus on nursing quality and comprise requirements that directly target the roles and competencies of nursing personnel, e.g. the Magnet Recognition Program^®^ [12], others do not explicitly focus on nursing but have relevance for nursing through mandates such as infection prevention and control [13]. To our knowledge, there is a lack of comprehensive overview or comparison of international accreditation/certification schemes with a focus on nursing in hospital settings, moreover, there is no comparative review that refers to nursing and quality of care indicators in such schemes.

Drawing on Donabedian’s quality dimensions, the effects on quality of the analyzed accreditation/certification schemes can be reflected in structure-, process- or outcome-related quality [14]. There is a broader range of research findings. A systematic literature review found positive effects of accreditations and certifications on safety culture, efficiency and processes [15]. A cross-sectional study showed positive associations with quality and safety in relation to the diseases considered [16]. Based on a systematic literature review, it was concluded that there are no effects of accreditation on employee satisfaction as well as patient satisfaction or experience [15]. In contrast, an analysis of secondary data of Magnet^®^-designated, Magnet^®^-in-progress and non-Magnet^®^ hospitals showed higher scores in patient satisfaction for Magnet^®^ hospitals [17]. Also, a survey of nurses in Magnet^®^ hospitals found higher satisfaction rates and better quality of patient care compared to hospitals recognized in the 1980s for their excellence in nursing (original Magnet^®^ hospitals) [18]. A literature review concluded findings of accreditations on patient outcomes to not be consistent [19]. Measuring effects in practice is methodologically challenging, due to the fact that the accreditation/certification schemes represent a complex intervention [9, 20]. Still, outcome indicators are of particular interest for consumers as well as health care payers [21]. Although the effects of accreditation/certification schemes have been analyzed on several outcome parameters, an overview on the usage of different outcome indicators, particularly the involvement of nurse-sensitive indicators, in the accreditation/certification processes has been largely missing.

Against this backdrop, this study investigates the following question: Which international accreditation/certification schemes exist for hospitals with relevance to nursing and take nursing quality into account? To answer the research question this study aims to identify international accreditation or certification schemes with a focus on or relevance to nursing in hospital settings (research aim I), analyze the conceptual models of quality assessment employed by these schemes, including its focus on or relevance for nursing quality (research aim II), and compare which outcome indicators are being used and related to nursing within these schemes (research aim III). In this study, schemes with explicit focus on nursing will be referred to as ‘nursing-specific’ and schemes with no explicit focus on nursing as ‘nursing-related’.

## Method

### Study design

A scoping review was conducted following the Joanna Briggs Institute methodology [22]. The scoping review was chosen as methodology due to its exploratory and broader approach in contrast to other review forms (ibid.). It allows to gather information on available accreditation/certification schemes for hospitals from different databases and types of publications (ibid.). A protocol was developed a priori and published (DOI 10.17605/OSF.IO/P548F) [23].

This study followed the Preferred Reporting Items for Systematic Reviews and Meta-Analyses extension for Scoping Reviews guideline (PRISMA-ScR) [24] (additional file 1).

### Search strategy

The search strategy was developed by the authors in collaboration with a librarian from the Technical University of Berlin. The search strategy was reviewed, adapted, and an exemplary application within one database conducted jointly between one author (CG) and the librarian. The final search strategy was discussed and agreed upon with the co-authors. Search terms were identified using the SPICE (setting, perspective, intervention, comparison, evaluation) framework [25]. The SPICE framework was chosen for its aspects ‘setting’ and ‘perspective’, highlighting the relevance to nursing within the accreditation/certification schemes. The term ‘evaluation’ instead of ‘outcome’ allowed for a broader research focus.

The final search covered the following terms: hospital, nurse, quality, improvement, performance, safety, excellence, accreditation, certification, designation, and distinction. The terms listed are free terms, which were supplemented by controlled language in each database whenever possible. Consequently, the search strategy was adjusted for each database. The applied search strategies are shown in the appendix (additional file 2).

The search was carried out in Medline, CINAHL, Web of Science Social Sciences Citation Index and Cochrane Reviews in January 2024. Additionally, the search was also run in Google Scholar to identify grey literature. Within Google Scholar, the first 100 search results were screened.

### Inclusion and exclusion criteria

Inclusion and exclusion criteria for studies were defined as follows.

Studies were included if they addressed international accreditation/certification schemes applicable to hospitals, irrespective of hospital location or characteristics, and had at least relevance to nursing (e.g. covering all staff, including nurses). The accreditation/certification schemes addressed in the studies had to aim at quality improvement of patient care, focus on the entire hospital (hospital-wide focus), be based on transparent criteria, be carried out by an external organization, and require a formal application process by the hospitals. In order for studies to be included, they had to contribute to answering at least one of the research aims, hence, presenting a scheme with relevance to nursing, providing information on the conceptual model of quality assessment and/or included outcome indicators. Studies published in English or German were included.

Excluded were studies in all other languages as were protocols, commentaries, opinion pieces, editorials, books or book chapters, conference abstracts and qualitative studies. Studies focusing on accreditation/certification schemes limited to a national context, revolving around outpatient/ambulatory care settings or specialty hospitals were excluded.

### Screening and analysis

After the application of the search strategy in the databases, all identified records were imported to Citavi and duplicates removed. The screening of records was based on inclusion and exclusion criteria, which were discussed in advance to ensure uniform understanding of the criteria. Records screening was split into two steps. First, titles and abstracts of the records were screened by two researchers independently (CG, JK). Inconsistencies between the researchers were discussed until consensus was reached. Second, full texts for the remaining records were retrieved and screened. Researchers split the review of full texts and data extraction after an intensive period of piloting to ensure consistency. For all included studies, information was extracted using a data chart (CG, JK). The following items were retrieved from the studies: Title, author(s), year of publication, aim of study, addressed accreditation/certification, accreditation/certification body, reported aim of the accreditation/certification, focus on nursing, conceptual information, additions/comments on concept, accreditations/certifications indicators on satisfaction of employees or patients, adverse events in patients, other data collection (e.g. key personnel figures), frequency of data collection, compulsoriness of data collection, and existence of benchmarking.

To extract the aspect ‘focus on nursing’ from the data, it was analysed whether the screened studies specifically addressed the nursing profession (nursing-specific schemes) or included all professions, encompassing nursing (nursing-related schemes). Schemes were identified as ‘nursing-specific’, if (a) the nursing workforce was explicitly the target group of the scheme, and/or (b) the quality assurance or improvement processes and indicators were specifically targeted at nurses (e.g. nurse-sensitive indicators). Schemes were categorized as ‘nursing-related’, if (a) nurses were (implicitly) of relevance, together with other professions, and/or (b) if quality assurance or improvement processes and indicators were broad in scope and were not specifically targeted at nurses, but all professions.

An assessment of the studies’ quality was not carried out, as the focus was on available information about accreditation/certification schemes, including their concepts and outcome indicators, rather than on comparing studies’ research results. Authoritative websites of accreditation/certification holders were reviewed for further information (CG). Aspects for schemes’ comparison were formed inductively.

## Results

### International accreditation and certification schemes

A total of 124 studies were included. Among these, 44 studies covered the concept of the schemes and 16 outcome indicators.

**Fig. 1:**
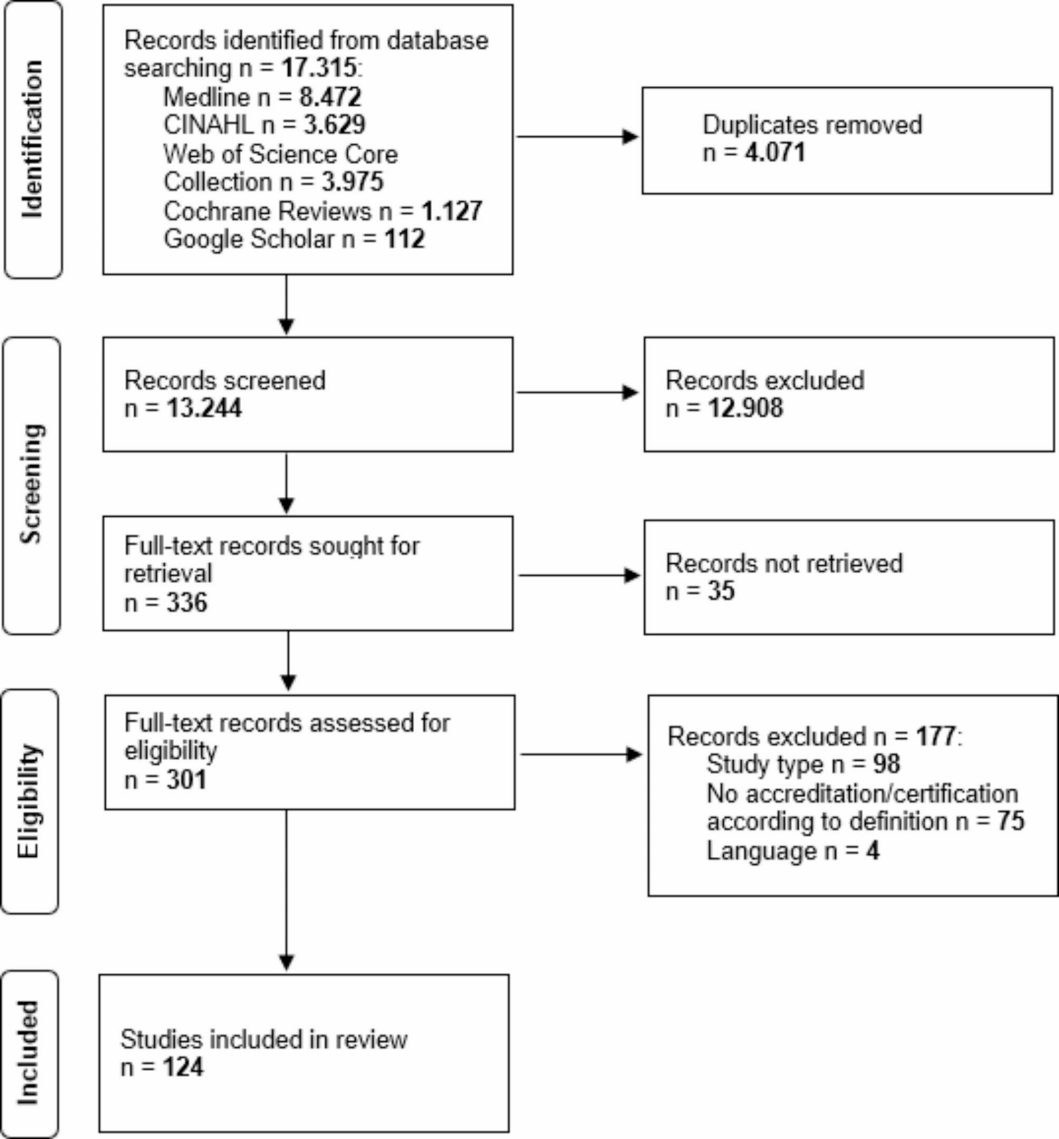
PRISMA-ScR flow diagram

The process and result of record screening and study inclusion are shown in the following figure (Fig. 1).

An overview of the included studies and their breakdown by scheme is attached in the appendix (additional file 3).

Seven international accreditation and certification schemes were identified (Table 1):

- European Foundation for Quality Management (EFQM)
- Evaluation and Quality Improvement Program (EQuIP)
- International Organization for Standardization (ISO) 9001
- Joint Commission International^®^ (JCI)
- Magnet Recognition Program^®^ (Magnet^®^)
- Pathway to Excellence^®^ Program (Pathway to Excellence^®^)
- Qmentum^®^

**Table 1:** Overview and main characteristics of seven identified international accreditation/certification schemes

	**EFQM**	**EQuIP**	**ISO 9001**	**JCI**	**Magnet®**	**Pathway to Excellence®**	**Qmentum®**
Accreditation/ certification body	European Foundation for Quality Management (EFQM) [26]	Australian Council on Healthcare Standards International (ACHSI) [27]	Various external certification bodies [28]	Joint Commission International [29]	American Nurses Credentialing Center (ANCC) [12]	American Nurses Credentialing Center (ANCC) [30]	Accreditation Canada [31]
Year of introduction of the international scheme	1989 [32]	2005 [27]	1987 [33]	1994 [34]	2000 [12]	2009 [35]	2008 [36]
Health care sector specific	No [32]	Yes [27]	No [37]	Yes [34]	Yes [12]	Yes [30]	Yes [31]
Overall aims	Enablement to manage change and improve performance [26]	Ensuring standards of quality as well as patient care [38]	Improvement of performance and demonstration of dedication to quality [37]	Ensuring patient safety [29]	Recognition of nursing excellence to improve patient outcomes [39]	Recognition of the commitment to build an engaging and empowering work environment for nurses [30]	Ensuring the safety and effectiveness of quality of care [40]
Accreditation/ certification levels	Three levels: - Validated - Qualified - Recognised [41]	No information	- [33]	4 classifications: - Accreditation with full standards compliance - Accreditation with recommen-dations for improvement - Provisional accreditation - Conditional accreditation [42]	Two levels: - Magnet® designation - Magnet® with distinction [39]	- [43]	Three levels: - Accredited (with report) - Accredited with commendation - Accredited with exemplary standing [44]
Validity period	3 years [45]	4 years [46]	3 years [33]	3 years [47]	4 years [48]	4 years [49]	4 years [44]
Number of accredited/ certified organizations	>50,000 [26]	No information	>1,000,000 [37]	1,029 [50]	591 [51]	214 [52]	>10,000 [40]

Key: “-“ = not applicable, “no information” = based on the retrieved information no statement can be made

*Abbreviations*: European Foundation for Quality Management (EFQM), Evaluation and Quality Improvement Program (EQuIP), International Organization for Standardization (ISO) 9001, Joint Commission international® (JCI), Magnet® Recognition Program (Magnet®), Pathway to Excellence® Program (Pathway to Excellence®)

The locus of these accreditation/certification bodies range from North America, Europe to Australia [12, 26–31]. The schemes were first applied internationally from 1987 to 2009 [12, 27, 32–36].

Five of the seven schemes are health care specific, exclusively addressing health care organizations [12, 27, 30, 31, 34], while EFQM and ISO 9001 do not specifically focus on the health sector and are used by the highest number of organizations in comparison to the other schemes [26, 32, 37].

Except for one, all have similar aims, namely to improve patient outcomes or performance [26, 29, 37–40]. In contrast, one scheme (Pathway to Excellence^®^) follows the aim to create a healthy work environment for nursing and thereby implicitly focuses on improving patient outcomes [30].

Each scheme requires a re-certification/-accreditation after three to four years [33, 44–49]. Additionally, EFQM, JCI, Magnet^®^ as well as Qmentum^®^ differentiate between various levels of accreditation/certification, which reflect differences in the quality of the accredited/certified organizations [39, 41, 42, 44].

### Conceptual model of quality assessment

The following sections provide a comparison of the schemes in terms of their thematic and nursing focus, use of structure, process and/or outcome quality as well as structure and content of their requirements (Table 2).

**Table 2:** Conceptual model of quality assessment for seven international accreditation/certification schemes

	**EFQM**	**EQuIP**	**ISO 9001**	**JCI**	**Magnet®**	**Pathway to Excellence®**	**Qmentum®**
**Focus on nursing**	Nursing-related (=all professions covered, including nurses) [26]	Nursing-related (=all professions covered, including nurses) [46]	Nursing-related (=all professions covered, including nurses) [33]	Nursing-related (=all professions covered, including nurses) [13]	Nursing-specific (=focus on the nursing professions, including nurse-sensitive quality and benchmarking) [53, 54]	Nursing-specific (=focus on the nursing profession, including working environments) [43, 55]	Nursing-related (=all professions covered, including nurses) [36, 56]
**Thematic focus **	Model for management [26]	Clinical, support and corporate functions of an organization [46]	Quality management [33]	Health care delivery with emphasis on patient safety [57]	Nursing Excellence [12]	Healthy working environment for nurses [43]	Broad scope of requirements regarding all aspects of the operation of an organization [56]
**Requirements focus on structure, process and/or outcome quality**	Structure, process and outcome quality [26]	Structure and process quality [46]	Process quality [33]	Process quality [57]	Structure and outcome quality [53]	No information	Structure and process quality [36]
**Structure and content of schemes’ requirements**	Seven criteria belonging to three topics: · Focus · Realization · Results Organizations are assessed against their individual objectives and processes. [26]	3 standard sets: · Clinical function · Support function · Corporate function In total 13 standards to meet [46]	Framework with requirements in seven topics: · Context of the organization · Leadership and accountability · Planning · Support · Operation · Evaluation of performance · Improvement Organizations are assessed against their individual objectives and processes. [33]	Standards divided into two categories: · Patient-centred care standards · Management of the health institution [13]	Five key components: · Transformational leadership · Structural empowerment · Exemplary professional practice · New knowledge, innovation, improvements · Empirical outcomes [53]	Six standards to fulfil: · Shared decision-making · Leadership · Safety · Quality · Well-being · Professional development [43]	Four mandatory standards to fulfil: · Governance · Leadership · Medication management · Infection prevention and control In total 100 standards related to eight quality dimensions, standards for assessment chosen by organization [36, 44, 56]

Key: “-“ = not applicable, “no information” = based on the retrieved information no statement can be made

*Abbreviations*: European Foundation for Quality Management (EFQM), Evaluation and Quality Improvement Program (EQuIP), International Organization for Standardization (ISO) 9001, Joint Commission international® (JCI), Magnet® Recognition Program (Magnet®), Pathway to Excellence® Program (Pathway to Excellence®)

#### Thematic and nursing focus

The accreditation/certification schemes follow different thematic foci when assessing a hospital’s quality, with varying focus on nursing.

EFQM is a model for management that supports organizations to enable continuous improvement [26]. It allows its usage for determining organizational excellence [58]. The requirements of ISO 9001 focus on an organization’s quality management [33]. It sets baseline requirements to meet customer expectations and product quality [33]. No professional groups are named in the frameworks of EFQM and ISO 9001. However, both frameworks expect employees to be involved in organizational structures and processes [26, 33]. Consequently, those schemes were identified as nursing-related instead of nursing-specific.

The accreditation by JCI focuses on the process of health care delivery with an emphasis on patient safety [57]. Qmentum^®^ comprises standards on all areas of running an organization [56], whereas EQuIP’s requirements focus on clinical, support and corporate functions [46]. JCI addresses all professional groups employed in a hospital, hence was categorized as relevant for nurses, but not nursing-specific [13]. For instance, requirements on infection prevention and control or coordination of transplantations and ‘staff qualifications and education’ cover all health professions, including nursing [13]. Research has addressed the role of nursing during accreditation and the impact of accreditation on the nursing work environment and documentation in patient files [42, 59, 60]. Within the available information for EQuIP and Qmentum^®^, nursing is not explicitly mentioned and instead, broader terms are used. Terms used include health staff that is addressed by and needed for fulfillment of the requirements [46, 56]. Moreover, Qmentum^®^ requires staff participation in a survey to evaluate work life, patient safety culture and governance [36]. Hence, those schemes were categorized as well as nursing-related.

While Magnet^®^ strives for and recognizes nursing excellence [12], Pathway to Excellence^®^ designates organization for healthy working environments for nurses [43]. Therefore, Magnet^®^ and Pathway to Excellence^®^ were both categorized as nursing-specific.

#### Structure, process, outcome quality

The accreditation/certification schemes differ in their emphasis on structure, process, and outcome quality. There is no available information regarding the emphasis of Pathway to Excellence® on structure, process, or outcome quality.

ISO 9001 and JCI are predominantly process-orientated [33, 57]. Two studies analyzed benefits of a certification by ISO and highlighted its process-orientated approach [61, 62].

Qmentum^®^ and EQuIP concentrate on the structure and process quality [36, 46]. An analysis of the evolution on the Magnet^®^ concept, using the three quality dimensions of Donabedian, underlined its focus on structure and outcome quality [63].

EFQM includes requirements on all three quality dimensions [26], including outcome measures of customers and employee orientation [58].

#### Structure and content of schemes requirements

EFQM and ISO 9001 are characterized by providing frameworks for quality improvement and assessment [26, 33]. Successful fulfillment of accreditation/certification requirements is defined individually for each organization, based on their objectives, processes, needs and provided services [26, 33]. The EFQM model consists of seven criteria that are divided into the themes ‘focus’, ‘realization’ and ‘results’ [26]. The high relevance of customer-related results and employee orientation are highlighted when comparing this scheme against others [58]. ISO 9001 criteria are divided into the topics ‘context of the organization’, ‘leadership and accountability’, ‘planning’, ‘support’, ‘operation’, ‘evaluation of performance’ and ‘improvement’ [33]. The certifications content and benefits were analyzed by several studies [58, 61, 62]. Nursing is not specifically mentioned within the requirements of either scheme; however, the criteria cover all professions.

In contrast, the other schemes contain specific requirements to be met.

EQuIP comprises 13 standards that are assigned to the three main topics ‘clinical function’, ‘support function’, ‘corporate function’. The first topic comprises standards such as ‘continuity of care’ or ‘access to care’ and are mainly to be fulfilled by health professionals [46]. The second includes standards such as ‘quality improvement and risk management’ or ‘human resources management’ and thereby involves health professionals and corporate staff for reaching the requirements [46]. The third topic covers the standards ‘leadership and management’ and ‘safe practice and environment’ whose fulfillment lies within the responsibility of the governing body [46]. Nursing is covered as one group of health professionals, though not mentioned explicitly.

JCI’s standards address a broad range of aspects of health care delivery such as ‘access to care and continuity of care’, ‘assessment of patients’ or ‘medication management and use’ [13]. The requirements of JCI are divided into ‘patient-centered standards’, ‘management standards’ and additional standards for academical hospitals [13]. Six studies explored the content and benefits of JCI and three studies analyzed the change of nursing documentation and medication errors pre and post accreditation [42, 60, 61, 64–69]. A study providing an overview of accreditation bodies concluded that all standards of JCI are directly or indirectly related to patient safety [42]. Through mandates such as infection prevention and control nursing is targeted by the requirements.

Qmentum^®^ comprises nearly 100 different standards that reflect all aspects of running an organization. Standards to include in the accreditation process need to be picked by hospitals with respect to their relevance for the individual hospitals. Though, the fulfillment of the following four standards is mandatory: ‘Governance’, ‘leadership’, ‘medication management’, ‘infection prevention and control’. As described above, nursing is also addressed by mandates such as infection control and prevention. Each standard is assigned to one of eight quality dimensions. The quality dimension ‘population health’ is of high relevance. Also, the accreditation’s inclusion of the dimensions ‘safety’ and ‘worklife’ is emphasized [36, 56].

The Magnet^®^ model focuses explicitly on nursing and comprises five components to foster nursing excellence: ‘Transformational leadership’, ‘structural empowerment’, ‘exemplary professional practice’, ‘new knowledge, innovation and improvements’ and ‘empirical quality results’ [53]. Magnet^®^ applies 14 forces of magnetism, each of which is assigned to one of the five components [53]. Nine of the included articles described the different Magnet^®^ components and forces of magnetism and provided examples of how to apply these in practice. Eight articles focused explicitly on the uptake of ‘new knowledge, innovation and improvements’ in practice and 13 articles set their focus on analyzing nurse satisfaction, patient satisfaction and/or required nurse-sensitive outcomes [17, 63, 70–97]. The importance of forming a Magnet^®^ culture for adapting the schemes’ requirements is mentioned [53]. Four of the included studies described the process of attaining a Magnet^®^ culture [98–101].

Pathway to Excellence^®^ is also oriented towards nursing and consists of six standards to promote a healthy working environment for nurses: ‘Shared decision-making’, ‘leadership’, ‘safety’, ‘quality’, ‘well-being’, ‘professional development’ [43]. The studies included focused on the uptake of the ‘professional development’ of nurses in practice [79, 82]. For achieving Pathway to Excellence^®^, organizations have to demonstrate their ability to enculture these standards [43].

### Outcome indicators

The seven international schemes show differences in the involved outcome indicators regarding the selection and inclusion of nursing quality, compulsoriness of data collection, use of external benchmarking and involvement of data results in the accreditation/certification process (Table 3).

**Table 3:** Included outcome indicators of seven international accreditation/certification schemes

	**EFQM**	**EQuIP**	**ISO 9001**	**JCI**	**Magnet®**	**Pathway to Excellence®**	**Qmentum®**
Performance indicators / adverse events	Performance measurements, organizations develop and select their own indicators [26]	Clinical Indicator Program (CIP), hospitals self-select relevant indicators from CIP [102]	- [33]	Patient outcomes, for instance safe use of opioids or influenza immunization (ORYX®), in addition to mandatory indicators, hospitals self-select relevant indicators [103]	Nurse-sensitive outcomes, for instance falls with injury or pressure ulcers, in addition to mandatory indicators. Hospitals self-select relevant indicators for data collection [54]	- [43]	Adverse events, near misses, hospitals self-select relevant indicators [104]
Frequency of data collection	- [26]	Semi-annual [102]	-	Quarterly [103]	Quarterly [54]	-	Regular (e.g. quarterly) [104]
Organizational outcomes	Financial and non-financial performance measurements, organizations develop and select their own indicators [26]	No information	- [33]	No information	Organizational outcomes in relation to nursing, for instance nurses’ certification [54]	No information	No information
Frequency of data collection	- [26]	No information	-	No information	No information	No information	No information
Quantitative data on employees´ opinion/ satisfaction	People’s perceptions [26]	No information	- [33]	No information	Nurse satisfaction [54]	Nurses perception of workplace environment [55]	Staff surveys about work life, patient safety culture, governance [36]
Frequency of data collection	- [26]	No information	-	No information	Once, within 30 months before starting designation process [54]	Once in order to be able to receive accreditation [55]	Minimum: once per accreditation cycle (4 years) [36]
Quantitative data on patients’ opinion/ satisfaction	Stakeholder perceptions [26]	Patient / consumer satisfaction [46]	Customer satisfaction [33]	No information	Patient satisfaction [54]	- [43]	Client experience survey [105]
Frequency of data collection	- [26]	No information	-	No information	Quarterly [54]	-	No information
Compulsory or voluntary data collection	Compulsory with restrictions [26]	No information	Compulsory with restrictions [33]	Compulsory [103]	Compulsory [54]	Compulsory [55]	Compulsory [36, 105]
Benchmarking	External Benchmarking [26]	External benchmarking [46]	- [33]	External benchmarking [106]	External benchmarking, hospitals are required to excel the benchmark mean for patient, nurse satisfaction and nurse-sensitive outcomes [54]	- [55]	External benchmarking [36]

Key: “-“ = not applicable, “no information” = based on the retrieved information no statements can be made

Abbreviations: European Foundation for Quality Management (EFQM), Evaluation and Quality Improvement Program (EQuIP), International Organization for Standardization (ISO) 9001, Joint Commission international® (JCI), Magnet® Recognition Program (Magnet®), Pathway to Excellence® Program (Pathway to Excellence®)

#### Performance indicators or adverse events

Five schemes, EFQM, EQuIP, JCI, Magnet^®^, Qmentum^®^, require data on adverse events, whereas ISO 9001 and Pathway to Excellence^®^ do not have such requirements [33, 43].

EFQM requires a measurement of the performance through appropriate indicators, selected by each organization [26]. Exemplary topics for such indicators are provided, but are neither exhaustive nor restrictive [26]. EFQM is not specifically tailored to health-related contexts; therefore, neither clinical indicators nor nurse-sensitive indicators are explicitly referenced, although their inclusion is not precluded. There is no specification for the frequency of data collection [26].

EQuIP includes the Clinical Indicator Program (CIP), providing organizations with a set of clinical indicators to select from [102]. Due to data restrictions, it cannot be assessed whether the clinical indicators comprise nurse-sensitive indicators. Data is to be sent in semi-annually for analysis and comparison [102].

While historically there was no focus on patient outcomes in JCI, two studies reported how patient indicators were included in the accreditation process over time [68, 69]. The initiative ORYX^®^ was founded to collect and evaluate patient outcomes [103]. In addition to mandatory patient outcomes, it is up to the hospital to decide which outcomes data are collected [103]. Examples of patient outcomes collected comprise a safe use of opioids, influenza immunization and cardiovascular or obstetric outcomes [103]. Based on the available data, nurse-sensitive indicators are not the primary focus and not mandatory to collect. However, it cannot be excluded that some nurse-sensitive indicators may be optionally reported. Four studies described the process and development of indicators collected, one study analyzed the change of medication errors during the accreditation journey [65, 66, 68, 69, 107]. A collection of the data is required quarterly [103].

For Magnet^®^ designation, data on nurse-sensitive outcomes has to be collected on a quarterly basis [54]. Inpatient indicators include falls with injury, plus three additional inpatient nurse-sensitive indicators for which data collection is to be based on relevance to practice, e.g. hospital-acquired pressure ulcers or central line bloodstream infection (ibid.). Two of the included studies analyzed differences in falls and central line bloodstream infections between Magnet^®^ and non-Magnet^®^ hospitals [89, 97].

For Qmentum^®^ accreditation, data needs to be collected on a regular basis for adverse events and near misses [104]. Hospitals self-select appropriate indicators for which data is sent in [104]. Based on the available data, it cannot be determined whether nurse-sensitive indicators are included.

#### Organizational outcomes

ISO 9001 does not require data on organizational outcomes and JCI includes organizational requirements, though it is unclear whether indicators on organizational outcomes are required [13]. Specific information on whether data on organizational outcomes is needed is only available for EFQM and Magnet^®^.

Similar to the performance evaluation, EFQM provides examples for assessing organizational outcomes, whereby organizations have to select/develop those being suitable [26]. Performance measurements should comprise financial and non-financial indicators [26]. Due to its generic approach, clinical or nurse-related indicators are not specified. However, as noted earlier, this does not imply the exclusion of these. No requirements for frequency of data collection are set [26].

Magnet^®^ requires organizational outcomes with relevance to nursing. This includes certification of registered nurses, among others [54].

#### Outcomes on patient experience and satisfaction

Four schemes, EFQM, ISO 9001, Magnet^®^, and Qmentum^®^, require outcomes on patient/customer experience or satisfaction. They largely differ in the thematic focus and ways of data collection.

EQuIP involves the evaluation of patient/consumer experience, but it remains unclear, how and by whom data is retrieved and whether nursing care experience is integrated into the assessment of patient/customer experience [46]. Information gathered for JCI does not address this topic. Pathway to Excellence^®^ does not require data on patients’ satisfaction [43].

EFQM and ISO 9001 require data on stakeholder perceptions and customer satisfaction [26, 33]. Both list several ways of collecting the data, for instance by conducting surveys, analyzing social media or the market share, without defining the regularity of data collection [26]. Their generic approaches do not provide specific requirements on whether the experience with nursing care should be collected or not.

Magnet^®^ and Qmentum^®^ require data on patient satisfaction, respectively customer experience. Data to be collected for conducting the survey is predefined by accredited vendors or standardized survey instruments. For Magnet^®^, the survey explicitly includes patient satisfaction with nursing. On Qmentum^®^, the inclusion of the nursing experience within the questionnaire is unclear [54, 105, 108]. Three studies examined patient satisfaction in Magnet^®^ hospitals or compared patient satisfaction within Magnet^®^-designated and non-Magnet^®^ hospitals [17, 88, 109]. While Magnet^®^ requires quarterly data results on patient’s satisfaction [54], the required frequency of data collection by Qmentum^®^ is not known.

#### Outcomes on staff satisfaction

Data on employee satisfaction is required by four (EFQM, Magnet^®^, Pathway to Excellence^®^, Qmentum^®^) of the seven schemes with differences in the group of people surveyed and the instruments for data collection.

ISO 9001 does not contain requirements for data collection on employees’ satisfaction [33]. For EQuIP and JCI no information was found on the requirement of quantitative data collection on employee’s opinion.

EFQM expects to gain insights on employees’ perceptions and suggests different thematic topics and sources to collect the data from, for instance through surveys, social media or by analyzing complaints [26]. The frequentness of data collection is not specified (ibid.).

Magnet^®^ and Pathway to Excellence^®^ focus on nurses’ satisfaction with their working environment [54, 55]. Seven studies analyzed the satisfaction rates of nurses within Magnet^®^ hospitals or between Magnet^®^ and non-Magnet^®^ hospitals [86, 87, 90, 91, 93, 95, 109]. The surveys’ contents are standardized [55, 108]. For Magnet^®^, several accredited vendors exist that offer surveys including the required benchmarking [108]. Magnet^®^ and Pathway to Excellence^®^ specify that the survey needs to be conducted once for the accreditation [54, 55].

Qmentum^®^ includes at least one staff survey on work life, patient safety culture as well as governance per accreditation cycle [36]. Instruments to be used are standardized (ibid.).

#### Compulsory vs. voluntary data collection

Collection of the above described data is compulsory for JCI, Magnet^®^, Pathway to Excellence^®^ and Qmentum^®^ [36, 54, 55, 103, 105].

It needs to considered that EFQM and ISO 9001 give several examples for data to be collected but do not specify mandatory data requirements [26, 33]. Therefore, it remains unclear, what data from which sources are accepted for accreditation/certification.

For EQuIP accreditation, it is unclear whether participation in CIP provides a benefit for accredited hospitals or if participation in CIP is mandatory for accreditation [102].

#### Benchmarking of data

Five of the schemes (EFQM, EQuIP, JCI, Magnet^®^, Qmentum^®^) involve external benchmarking, giving organizations the possibility of or the mandatory requirement to compare themselves to other organizations and identify room for improvement [26, 36, 46, 54, 106].

ISO 9001 and Pathway to Excellence^®^ do not require external benchmarking [33, 55].

Magnet^®^ is the only scheme that includes hospital benchmarking in the designation decision. Hospitals applying for designation are required to achieve better results over eight quarters in patient satisfaction and nurse-sensitive outcomes compared to the benchmark mean [54]. Additionally, nurse satisfaction must exceed the benchmark mean, and the survey must be no older than 30 months when applying for Magnet® designation (ibid.).

## Discussion

This scoping review identified seven international accreditation or certification schemes with relevance to nursing: EFQM, EQuIP, ISO 9001, JCI, Magnet^®^, Pathway to Excellence^®^, Qmentum^®^. The schemes ranged from nursing-specific (two schemes: Magnet^®^, Pathway to Excellence^®^) to no specific focus on nursing but with relevance for nurses (five schemes: EFQM, EQuIP, ISO 9001, JCI, Qmentum^®^). The schemes subsequently had different thematic foci, and different emphases on structure, process and outcome quality measures. Additionally, the types of outcome indicators required varied significantly across the schemes. Differences concerned the selection of outcome indicators, including nurse-sensitive indicators, the compulsoriness of data collection and the use of external benchmarking.

The emphasis on nursing in our study reflects the critical role nurses play in delivering patient care [3], including the fact that nurses are usually the only profession being constantly on wards with patients. Therefore, measuring nursing quality in quality assurance would be a way to make the contribution of nurses visible. However, it is equally essential to recognize the contributions of all professional groups in hospital settings. While schemes like Magnet^®^ and Pathway to Excellence^®^ specifically target nursing [43, 53], other schemes covered all healthcare professionals, including physicians, nurses, allied health professionals, administrative staff, and support staff [13, 26, 33, 56, 102]. A cross-sectional study in Denmark investigated attitudes of Danish hospital managers, physicians, nurses and staff responsible for quality improvement towards hospital accreditation [110]. The survey results showed overall positive attitudes towards accreditation, with physicians being more skeptical in contrast to the other professional groups (ibid.). The study concluded the importance of taking healthcare professionals’ attitudes for a successful implementation of quality improvement and accreditation into account (ibid.). A cross-sectional study in Iran, consisting of a survey to nurses, managers, administrative and para-clinical staff in Iranian hospitals, analysed the attitudes towards impact and benefits of accreditation [111]. Overall, the support for accreditation was low, highlighting the need for more involvement of healthcare professionals to understand the failure or success of accreditations (ibid.).

All schemes aim, at least indirectly, at improving quality of care and provide different concepts to do so. The diversity in thematic foci among the schemes, ranging from addressing the management, nursing, the quality management, to the process of healthcare delivery and general processes for running a health care organization [12, 26, 33, 43, 46, 56, 57], explains the co-existence of the schemes.

The root causes of patient safety have been attributed to the structure, culture, and processes of health care organizations [4, 112]. The schemes do address the structure and/ or process quality [26, 33, 46, 53, 56, 57]. Organizational culture has been recognized as an important aspect of health care outcomes [112], being integrated in all schemes [13, 26, 33, 40, 43, 46, 53].

The comparison of the schemes’ requirements was aligned with the level of available information. The requirements’ focus on nursing care was apparent within Magnet^®^ and Pathway to Excellence^®^. Both of these schemes are nursing-specific [12, 43]. In contrast, based on the available data, the requirements of the other five schemes are formulated in broader terms [13, 26, 33, 46, 56]. While nursing can be included in these schemes through mandates like infection control and prevention, it is not explicitly mentioned or a primary focus. A more detailed comparison of the assessed items that form the basis of these requirements could enhance understanding to what extent the schemes involve nursing quality.

Magnet^®^ and Pathway to Excellence^®^ include the requirements ‘Exemplary Professional Practice’ and ‘New Knowledge, Innovation, & Improvements’ respectively ‘Professional Development’, with the aim of fostering continuous learning for nurses, integrating new knowledge into patient care and providing high quality of care [43, 53]. Hence, both models impact professional development opportunities for nurses through their requirements. A secondary analysis of survey data from nurses in four states of the United States showed that nurses in Magnet^®^ hospitals were more educated compared to those in non-Magnet hospitals [113]. A study investigating the relationship between Magnet^®^ components and nurses job satisfaction in two European countries, using a cross-sectional survey of registered nurses, found that professional development significantly affected nurses job satisfaction [114]. Additionally, patient safety can be improved, as a cross-sectional study based on the National Database of Nursing Quality Indicators revealed that Magnet hospitals having a 5% lower fall rate than non-Magnet hospitals [89]. In contrast, within EFQM, EQuIP, JCI and Qmentum^®^ continuous learning and professional development of hospital staff is mentioned, but it is not elaborated upon in the available information [13, 26, 40, 46]. For ISO 9001, the role of professional development is not mentioned [33]. A systematic review on the effects of accreditation and certification status on quality of care highlights a lack of focus on the involvement of hospital staff in implementing the models’ requirements, indicating the need for more comparative research on the role of hospital staff, including nurses, in implementing accreditation or certification models and their effects on hospitals staff professional development [19].

Some schemes refer to demonstrating excellence such as ‘nursing excellence’ by Magnet^®^, ‘culture of sustained excellence’ by Pathway to Excellence^®^, ‘performance excellence’ by JCI, ‘excellence in patient care’ by EQuIP or, when considering EFQM, ‘organizational excellence’ [12, 43, 46, 57, 58]. The concept of excellence is gaining increased attention, indicating a shift from meeting minimum quality requirements to exceeding them [115]. A scoping review analyzed the concept of ‘Centres of Excellence’ [115]. These are defined by specialized expertise and providing better results than the average. It was found that the term ‘excellence’ is related to processes rather than outcomes (ibid.). Against the backdrop of limited financial resources for health care services [116], further, in-depth analysis of the term ‘excellence’ for hospital quality and the identification of excellent hospitals should be performed in future research.

All schemes include outcome indicators for which hospitals need to collect data in order to gain accreditation/certification [26, 33, 46, 54, 55, 103, 105]. Indicator validity and data quality differ [117]. When considering adverse events or performance indicators, the schemes require hospitals to self-develop indicators or self-select indicators from a list of predefined indicators [26, 38, 54, 103, 104]. Magnet^®^ places an emphasis on nurse-sensitive indicators [54]. However, due to limited information availability, it remains unclear whether nurse-sensitive indicators are required for EQuIP, JCI, and Qmentum^®^ [102–104]. EFQM, not being healthcare specific, does not require nurse-sensitive indicators, but organizations have the option to include them at their discretion [26]. Due to different levels of specializations of hospitals, the relevance of indicators can vary, hence, the self-selection of indicators allows hospitals to choose the ones most relevant for their organization. A possible disadvantage could be that hospitals self-select those indicators for which good results already exist.

When gathering data on patient experience or satisfaction, Magnet^®^ requires specifically data on the experience with nursing care [54]. It remains unclear whether and to what extent aspects of nursing care are incorporated into the questionnaires of EQuIP and Qmentum^®^ [46, 105]. With regard to data on patient or employee satisfaction, EFQM and ISO 9001 do not include requirements on content to be included or survey instruments [26, 33]. As a result, hospitals may utilize different data sources, exhibit varying satisfaction rates and experiences, yet still achieve the same accreditation/certification. This can contribute to the reduced transparency of the informative value of accreditation/certification schemes.

The Pathway to Excellence^®^ program sets requirements for the results of the nursing survey, while it is well established in the Magnet^®^ program that results on patient satisfaction, nurse satisfaction and nurse-sensitive indicators must surpass the benchmark mean to attain Magnet^®^ status [54, 118]. An external benchmarking adds to the transparency of accreditations/certifications results as results are put into relation with other hospitals. Yet, not all accreditation/certifications include a comparison to other organizations/hospitals results and apart from Magnet^®^ and Pathway to Excellence^®^ there is no knowledge about whether negative results on outcome indicators prevent accreditation/certification.

A study across five countries analyzed the quality and safety of European hospitals by comparing data availability and the definition of process- and outcome-related indicators for hospitals [11]. The study found that differences in the usage and definition of quality indicators as well as compulsoriness of data collection hinder the comparison of hospitals and prevents patients from making informed decisions on which hospital to select for their treatment (ibid.). Our comparison of outcome indicators confirmed the variety in the usage and definition across the schemes. It may be a challenge for patients, the public, policymakers and hospital managers to fully understand the value of the schemes.

Overall, the comparison of the schemes provides insights for nursing management. With the understanding that the focus on nursing quality varies significantly between schemes, as reflected in their conceptual models and outcome indicators, it is important when selecting a scheme to ensure that nursing quality is incorporated adequately. For enhancing healthcare quality and patient safety, the multidimensionality of quality needs to be taken into account.

This study faces the following limitations. Studies in languages other than English or German were not included, hence relevant studies may have been missed. Moreover, only publicly available information was used for this study. There was heterogeneity in the information publicly available by scheme, which hampered comparisons across the schemes, particularly on the items belonging to the requirements for accreditation/certification and required indicators.

This review provided an in-depth comparison of the schemes in their overall purpose, concepts and outcome indicators, yet, it did not analyze the comparative effectiveness of the schemes. Future research should synthesize existing studies on the effects of the schemes on quality for each scheme individually and across schemes. The involvement and professional development of hospital staff in implementing the accreditation and certification requirements as well as their acceptance of accreditation and certification schemes should also be investigated in further research.

## Conclusion

The scoping review identified seven accreditation and certification schemes with two schemes being nursing-specific and five schemes having relevance for nursing. Large differences exist in their conceptual models for quality assessment and outcome indicators. In their concept of quality assessment, nursing-specific schemes prioritized nursing, whereas nursing-related schemes generally adopted a broader quality perspective. With regards to the included outcome indicators, one scheme focused on nurse-sensitive indicators, while the others did not require data collection on nurse-sensitive indicators or did not emphasize clinical quality indicators. There were variations in the requirements of data collection on patient experience or satisfaction and staff satisfaction, compulsoriness of data collection and integration of benchmarking. Hospital and nursing managers need to understand the focus of different schemes when deciding which scheme to pursue. Understanding the schemes’ informative value is critical for patients, policymakers, and the public when assessing hospital quality.

## Supplementary Information

Supplementary Material 1.

Supplementary Material 2.

Supplementary Material 3.

## Data Availability

All data analyzed during this study are included in this published article and its supplementary information files.
