# Supplementary material for: International models of accreditation and certification for hospitals with a focus on nursing: a scoping review
Source: BMC Health Serv Res. 2024 Nov 12;24:1385. doi: 10.1186/s12913-024-11759-6 (PMC11559163; doi:10.1186/s12913-024-11759-6)
Supplement: Supplementary file 2 — Supplementary Material 2. [file 12913_2024_11759_MOESM2_ESM.docx]

**Additional file 2: Search strategy**

**Medline,** search carried out 19^th^ of January 2024

(("Hospitals"[MeSH Terms] OR "hospital*"[All Fields]) AND

("Nurses"[MeSH Terms] OR "nurs*"[All Fields]) AND

("quality*"[All Fields] OR "Quality of Health Care"[MeSH Terms] OR "Quality Improvement"[MeSH Terms] OR "improv*"[All Fields] OR "perform*"[All Fields] OR "excellen*"[All Fields] OR "Patient Safety"[MeSH Terms] OR "safety*"[All Fields]) AND

("Accreditation"[MeSH Terms] OR "accredit*"[All Fields] OR "Certification"[MeSH Terms] OR "certifi*"[All Fields] OR "designat*"[All Fields] OR "distinct*"[All Fields])) AND

(english[Filter] OR german[Filter])

**CINAHL**, search carried out 18^th^ of January 2024

((MH “Hospitals”) OR (hospital*)) AND

((MH ”Nurses”) OR (nurs*)) AND

((MH “Quality of Health Care) OR (quality*) OR (MH “Quality Improvement”) OR (improv*) OR (excellen*) OR (MH “Patient Safety”) OR (safety*) OR (perform*)) AND

((MH “Accreditation”) OR (MH “Magnet Hospital Accreditation”) OR (MH “American Accreditation Healthcare Commission) OR (MH “Joint Commission”) OR (accredit*) OR (MH “Certification”) OR (certifi*) OR (designat*) OR (distinct*))

Language: English, German

**Web of Science Social Sciences Citation Index**, search carried out 19^th^ of January 2024

(ALL=(hospital*) OR KP=(“hospital”)) AND

(ALL=(nurs*) OR KP=(“nurse”)) AND

(ALL=(quality*) OR KP=(“quality of healthcare”) OR ALL=(improv*) OR KP=(“quality improvement”) OR ALL=(perform*) OR ALL=(excellen*) OR ALL=(safety*) OR KP=(“patient safety”)) AND

(ALL=(accredit*) OR KP=(“accreditation”) OR ALL=(certifi*) OR KP=(“certification”) OR ALL=(designat*) OR ALL=(distinct*))

Languages: English, German

Excluded article types: Editorial Material, Book Chapters, Meeting Abstracts, Letter, Note, Book Review, Meeting

**Cochrane Reviews**, search carried out 18^th^ of January 2024

(hospital* OR MeSH descriptor: [Hospitals] explode all trees) AND

(nurs* OR MeSH descriptor: [Nurses] explode all trees) AND

(quality* OR MeSH descriptor: [Quality Assurance, Health Care] explode all trees OR improv* MeSH descriptor: [Quality Improvement] explode all trees OR perform* OR excellen* OR safety* MeSH descriptor: [Patient Safety]) AND

(accredit* MeSH descriptor: [Accreditation] explode all trees OR certifi* OR MeSH descriptor: [Certification] explode all trees OR designat* OR distinct*)

**Google Scholar**, search carried out 19^th^ of January 2024

Search string I: Hospital AND nursing AND quality AND accreditation

Search string II: Hospital AND nursing AND quality AND certification

Search string III: Hospital AND quality AND accreditation

Seach string IV: Hospital AND quality AND certification
