# Supplementary material for: International models of accreditation and certification for hospitals with a focus on nursing: a scoping review
Source: BMC Health Serv Res. 2024 Nov 12;24:1385. doi: 10.1186/s12913-024-11759-6 (PMC11559163; doi:10.1186/s12913-024-11759-6)
Supplement: Supplementary file 3 — Supplementary Material 3. [file 12913_2024_11759_MOESM3_ESM.docx]

**Additional file 3: Overview of included studies (n=124)**

| **Author(s)** | | **Year** | | **Title** | **Relevance for research aim 1: Name of certification/ accreditation** | **Relevance for research aim 2:  conceptual model of quality assessment** | **Relevance for research aim 3:  outcome indicators** |  |
| --- | --- | --- | --- | --- | --- | --- | --- | --- |
| Abolfotouh et al. | | 2014 | | Nursing Perception Towards Impact of JCI Accreditation and Quality of Care in a Tertiary Care Hospital, Central Saudi Arabia | JCI |  |  |  |
| Aiken et al. | | 2008 | | Transformative impact of Magnet designation: England case study | Magnet® | x | x |  |
| Aiken et al. | | 2009 | | The Magnet Nursing Services Recognition Program: A Comparison of Two Groups of Magnet Hospitals | Magnet® |  |  |  |
| Algahtani et al. | | 2017 | | Perception of hospital accreditation among health professionals in Saudi Arabia | JCI |  |  |  |
| Al-Qahtani et al. | | 2012 | | Quality of care in accredited and nonaccredited hospitals: perceptions of nurses in the Eastern Province, Saudi Arabia | JCI |  |  |  |
| Al-Sayedahmed et al. | | 2021 | | Impact of Accreditation Certification on Improving Healthcare Quality and Patient Safety at Johns Hopkins Aramco Healthcare | JCI |  |  |  |
| Al Shammari et al. | | 2015 | | Impact of hospital accreditation on patient safety in Hail city, Saudi Arabia: nurses’ perspective | JCI |  |  |  |
| Al Shawan | | 2021 | | The Effectiveness of the Joint Commission International Accreditation in Improving Quality at King Fahd University Hospital, Saudi Arabia: A Mixed Methods Approach | JCI |  |  |  |
| Anderson et al. | | 2018 | | Impact of MAGNET hospital designation on nursing culture: an integrative review | Magnet® | x |  |  |
| Auditore et al. | | 2017 | | Using the Magnet® Model to Develop a State of Nursing Report | Magnet® | x |  |  |
| Avia & Hariyati | | 2019 | | Impact of hospital accreditation on quality of care: A literature review | JCI |  |  |  |
| Barnes et al. | | 2016 | | Magnet® Hospital Recognition Linked to Lower Central Line-Associated Bloodstream Infection Rates | Magnet® |  | x |  |
| Batista et al. | | 2021 | | Authentic leadership, nurse satisfaction at work and hospital accreditation: study in a private hospital network | JCI |  |  |  |
| Beckel et al. | | 2013 | | Identification of potential barriers to nurse-sensitive outcome demonstration | Magnet® | x |  |  |
| Bilyeu et al. | | 2023 | | Magnet® and the charge nurse role | Magnet® |  |  |  |
| Blackwell | | 2020 | | Demonstrating Nursing Excellence Through Equality: The Relationship Between Magnet® Status and Organizational LGBTQ Client Services and Support | Magnet® |  |  |  |
| Bliss-Holtz et al. | | 2004 | | An invitation to Magnet accreditation | Magnet® | x |  |  |
| Bolton & Goodenough | | 2003 | | A magnet nursing service approach to nursing's role in quality improvement | Magnet® | x |  |  |
|  |  |  |  |  | JCI |  |  |  |
| Bord et al. | | 2021 | | Involvement and skepticism towards the JCI Accreditation process among hospital's four sectors employees: suggestions for cultural change | JCI |  |  |  |
| Boylan et al. | | 2019 | | Association of Magnet Nursing Status With Hospital Performance on Nationwide Quality Metrics | Magnet® |  |  |  |
| Broom & Tilbury | | 2007 | | Magnet status: a journey, not a destination | Magnet® | x |  |  |
| Buchan | | 1999 | | Still attractive after all these years? Magnet hospitals in a changing health care environment | Magnet® |  |  |  |
| Catalano | | 2008 | | The Joint Commission update for peri-operative services-2009 | JCI | x | x |  |
| Cimiotti et al. | | 2005 | | The magnet process and the perceived work environment of nurses | Magnet® | x | x |  |
| Clavelle & Goodwin | | 2016 | | The Center for Nursing Excellence: A Health System Model for Intentional Improvement and Innovation | Magnet® |  |  |  |
| Cosme et al. | | 2021 | | MAGNET® PERSPECTIVES. A Framework for Nursing Excellence | Magnet® | x |  |  |
| Devkaran & O'Farrell | | 2015 | | The impact of hospital accreditation on quality measures: an interrupted time series analysis | JCI |  |  |  |
| Devkaran et al. | | 2019 | | Impact of repeated hospital accreditation surveys on quality and reliability, an 8-year interrupted time series analysis | JCI |  |  |  |
| Dierkes | | 2021 | | The Association of Hospital Magnet ® Status and Pay-for-Performance Penalties | Magnet® |  |  |  |
| Drenkard | | 2005 | | Sustaining Magnet: Keeping the Forces Alive | Magnet® | x |  |  |
| Drenkard | | 2010 | | The Business Case for Magnet® | Magnet® |  |  |  |
| Drenkard | | 2013 | | The Value of Magnet® | Magnet® |  |  |  |
| Drenkard | | 2022 | | The Business Case for Magnet® Designation: Using Data to Support Strategy | Magnet® |  |  |  |
| Elkins et al. | | 2010 | | Perceived stress among nursing and administration staff related to accreditation | JCI |  |  |  |
| El-khateeb et al. | | 2022 | | Using Magnet Components in Nursing Practice | Magnet® | x |  |  |
| Evrenol Öcal & Terzioglu | | 2022 | | Determining the relationship between magnet properties of hospitals and the professional values of the nurses: A cross-sectional study | Magnet® |  |  |  |
|  |  |  |  |  | JCI |  |  |  |
| February & Holmes | | 2020 | | The Business Case for Magnet® in an International Hospital | Magnet® |  |  |  |
| Gonzalez et al. | | 2015 | | Impact of Magnet Culture in Maintaining Quality Outcomes During Periods of Organizational Transition | Magnet® | x | x |  |
| Goode et al. | | 2011 | | Comparison of patient outcomes in Magnet® and non-Magnet hospitals | Magnet® | x |  |  |
| Grant et al. | | 2012 | | Creating an innovative tool to measure Magnet® readiness | Magnet® |  |  |  |
| Guerra Bretana & Marin | | 2017 | | Accreditation and certification of hospital quality: different or similar | ISO 9001 | x |  |  |
|  |  |  |  |  | EFQM |  |  |  |
|  |  |  |  |  | JCI |  |  |  |
| Halasa et al. | | 2015 | | Value and impact of international hospital accreditation: a case study from Jordan | JCI |  |  |  |
| Havens | | 2001 | | Comparing nursing infrastructure and outcomes: ANCC magnet and nonmagnet CNEs report. | Magnet® |  |  |  |
| Hickey et al. | | 2014 | | Credentialing: the need for a national research agenda | Magnet® |  |  |  |
| Higdon et al. | | 2013 | | Business case for Magnet® in a small hospital | Magnet® |  |  |  |
| Houston & Miller | | 1997 | | The quality and outcomes management connection | JCI |  | x |  |
| Huseman-Maratea et al. | | 2022 | | Application of the Donabedian Model to Guide Virtual Magnet® Site Visit Preparations During a Pandemic | Magnet® |  |  |  |
| Jakubic et al. | | 2011 | | Nurse Mentoring Study Demonstrates a Magnetic Work Environment: Predictors of Mentoring Benefits Among Pediatric Nurses | Magnet® | x |  |  |
| Jenkins & Fields | | 2011 | | Pursuing Magnet® designation as a system or as individual entities: what is the right decision? | Magnet® |  |  |  |
| Johantgen et al. | | 2017 | | Building Research Infrastructure in Magnet® Hospitals: Current Status and Future Directions | Magnet® | x |  |  |
| Kagan et al. | | 2016 | | Effect of Joint Commission International Accreditation on the Nursing Work Environment in a Tertiary Medical Center | JCI | x |  |  |
| Karamali et al. | | 2020 | | Knowledge mapping of hospital accreditation research: a coword analysis | JCI |  |  |  |
| Karim et al. | | 2018 | | The Effect of the Magnet Recognition Signal on Hospital Financial Performance | Magnet® |  |  |  |
| Karkos & Peters | | 2006 | | A Magnet community hospital: Fewer barriers to nursing research utilization | Magnet® | x |  |  |
| Kramer et al. | | 2011 | | Clinical nurses in Magnet hospitals confirm productive, healthy unit work environments | Magnet® | x | x |  |
| Kramer & Schmalenberg | | 2005 | | Best quality patient care: a historical perspective on Magnet hospitals | Magnet® | x |  |  |
| K.S. et al. | | 2020 | | Impact of accreditation on performance of healthcare organizations | JCI |  |  |  |
|  |  |  |  |  | ISO 9001 |  |  |  |
| Lake et al. | | 2010 | | Patient falls: association with hospital magnet status and nursing unit staffing | Magnet® | x | x |  |
| Lake et al. | | 2012 | | Association between hospital recognition for nursing excellence and outcomes of very low-birth-weight infants | Magnet® |  |  |  |
| Lam et al. | | 2018 | | Association between patient outcomes and accreditation in US hospitals: observational study | JCI |  |  |  |
| Lasater & Schlak | | 2020 | | Quality of End of Life Care in Magnet® and Non-Magnet Hospitals | Magnet® |  |  |  |
| Lindlbauer et al. | | 2016 | | Changes in technical efficiency after quality management certification: A DEA approach using difference-in-difference estimation with genetic matching in the hospital industry | ISO 9001 |  |  |  |
| Malloch | | 2009 | | Living excellence: life after Magnet designation | Magnet® | x |  |  |
| Mansour et al. | | 2020 | | The development of hospital accreditation in low- and middle-income countries: a literature review | JCI |  |  |  |
| McCaughey et al. | | 2018 | | Magnetic work environments: Patient experience outcomes in Magnet versus non-Magnet hospitals | Magnet® | x | x |  |
| McGreevey et al. | | 1997 | | The Joint Commission on Accreditation of Healthcare Organizations' Indicator Measurement System. Health care outcomes database | JCI | x | x |  |
| McHugh et al. | | 2013 | | Lower mortality in Magnet hospitals | Magnet® |  |  |  |
| McLaughlin & Fetzer | | 2015 | | The perceived value of certification by Magnet® and non-Magnet nurses | Magnet® |  |  |  |
| Mehta et al. | | 2021 | | Assessment of Magnet status and Textbook Outcomes among medicare beneficiaries undergoing hepato-pancreatic surgery for cancer | Magnet® |  |  |  |
| Mensik et al. | | 2011 | | Development of a Professional Nursing Framework: the journey toward nursing excellence | Magnet® |  |  |  |
| Meredith et al. | | 2010 | | Transformational Leadership: Application of Magnet’s New Empiric Outcomes | Magnet® | x |  |  |
| Moffet et al. | | 2005 | | Strategic opportunities in the oversight of the U.S. hospital accreditation system | JCI |  |  |  |
| Moffet & Bohara | | 2005 | | Hospital quality oversight by the Joint Commission on the Accreditation of Healthcare Organizations | JCI |  |  |  |
| Muller et al. | | 2010 | | Sustaining excellence: clinical nurse specialist practice and magnet designation | Magnet® |  | x |  |
| Munroe & Lash | | 2005 | | Achieving Magnet recognition: the process | Magnet® |  |  |  |
| Muri | | 1998 | | The Joint Commission’s ORYX initiative: implications for perinatal nursing and care | JCI | x | x |  |
| Nelson-Brantley et al. | | 2020 | | Magnet® and Pathway to Excellence®: Focusing on Research and Evidence-Based Practice | Magnet® | x |  |  |
|  |  |  |  |  | Pathway to Excellence® |  |  |  |
| Nomura et al. | | 2016 | | Quality of nursing documentation before and after the Hospital Accreditation in a university hospital | JCI | x |  |  |
| Nomura et al. | | 2017 | | Quality of Electronic Nursing Records: The Impact of Educational Interventions During a Hospital Accreditation Process | JCI | x |  |  |
| Odundo et al. | | 2015 | | The Impact of ISO Certification of Healthcare Services on Complaints and Litigation—A Children’s Hospital’s Perspective | ISO 9001 |  |  |  |
| Patterson | | 1990 | | Quality assurance, control, and monitoring. The future role of information technology from the Joint Commission's perspective | JCI |  |  |  |
| Petit Dit Dariel & Regnaux | | 2015 | | Do Magnet®-accredited hospitals show improvements in nurse and patient outcomes compared to non-Magnet hospitals: a systematic review | Magnet® |  |  |  |
| Pintz et al. | | 2018 | | National Study of Nursing Research Characteristics at Magnet®-Designated Hospitals | Magnet® | x |  |  |
| Poduska | | 2005 | | Magnet designation in a community hospital | Magnet® | x |  |  |
| Ratcliffe | | 2009 | | Re‐engineering hospital accreditation | JCI |  |  |  |
|  |  |  |  |  |  |  |  |  |
| Robinson | | 2001 | | Magnet nursing services recognition: transforming the critical care environment | Magnet® | x |  |  |
|  |  |  |  |  | JCI |  |  |  |
| Sack et al. | | 2011 | | Is there an association between hospital accreditation and patient satisfaction with hospital care? A survey of 37,000 patients treated by 73 hospitals | JCI |  |  |  |
|  |  |  |  |  | ISO 9001 |  |  |  |
| Saufl & Fieldus | | 2003 | | Accreditation: a “voluntary” regulatory requirement | JCI | x |  |  |
| Schmaltz et al. | | 2011 | | Hospital performance trends on national quality measures and the association with Joint Commission accreditation | JCI |  |  |  |
| Selbmann | | 2004 | | Assessment and certification of hospital care in Germany | EFQM |  |  |  |
|  |  |  |  |  | JCI |  |  |  |
|  |  |  |  |  | ISO 9001 |  |  |  |
| Shaw et al. | | 2010 | | Accreditation and ISO certification: do they explain differences in quality management in European hospitals? | ISO 9001 |  |  |  |
| Shaw et al. | | 2014 | | The effect of certification and accreditation on quality management in 4 clinical services in 73 European hospitals | ISO 9001 |  |  |  |
| Sherman & Malkmus | | 1994 | | INTEGRATING QUALITY ASSURANCE AND TOTAL QUALITY MANAGEMENT QUALITY IMPROVEMENT | JCI |  |  |  |
| Shirey | | 2005 | | Celebrating certification in nursing: forces of magnetism in action | Magnet® | x |  |  |
| Silver | | 2004 | | Implications for librarians of magnet hospital designation | Magnet® |  |  |  |
| Smith | | 2003 | | Making the Magnet commitment | Magnet® |  |  |  |
| Smith | | 2014 | | Magnet Hospitals: Higher Rates of Patient Satisfaction | Magnet® | x | x |  |
| Spaulding et al. | | 2020 | | Do Magnet®-Designated Hospitals Perform Better on Medicare's Value-Based Purchasing Program? | Magnet® |  |  |  |
| Steinbinder | | 2009 | | Bumps on the road to Magnet designation: achieving organizational excellence | Magnet® |  |  |  |
| Stoimenova et al. | | 2014 | | ISO 9001 certification for hospitals in Bulgaria: does it help service? | ISO 9001 | x |  |  |
| Stone et al. | | 2019 | | Benchmarking nurse outcomes in Australian Magnet® hospitals: cross-sectional survey | Magnet® |  |  |  |
| Thornlow & Merwin | | 2009 | | Managing to improve quality: The relationship between accreditation standards, safety practices, and patient outcomes | JCI |  |  |  |
| Trinkoff et al. | | 2010 | | A Comparison of Working Conditions Among Nurses in MagnetA and Non-MagnetA Hospitals | Magnet | x | x |  |
| Tropello | | 2003 | | Magnet status as a competitive strategy of hospital organizations: marketing a culture of excellence in nursing services | Magnet® |  |  |  |
|  |  |  |  |  | JCI |  |  |  |
| Tuazon | | 2007 | | Is Magnet a money-maker? | Magnet® |  |  |  |
| Tubbs-Coley et al. | | 2017 | | Hospital Magnet® Designation and Missed Nursing Care in Neonatal Intensive Care Units | Magnet® |  |  |  |
| Ulrich et al. | | 2007 | | Critical care nurses’ work environments value of excellence in beacon units and magnet organizations | Magnet® | x | x |  |
| Vallejo et al. | | 2011 | | A Comparison of Hospital Accreditation Programs | JCI | x |  |  |
|  |  |  |  |  |  |  |  |  |
| van Bogaert et al. | | 2018 | | Staff empowerment and engagement in a magnet® recognized and joint commission international accredited academic centre in Belgium: a cross-sectional survey | JCI |  |  |  |
|  |  |  |  |  | Magnet® |  |  |  |
|  |  |  |  |  | ISO 9001 |  |  |  |
| van Wilder et al. | | 2021 | | Is a hospital quality policy based on a triad of accreditation, public reporting and inspection evidence-based? A narrative review | JCI |  |  |  |
|  |  |  |  |  | Magnet® |  |  |  |
|  |  |  |  |  | ISO 9001 |  |  |  |
| Vartanian et al. | | 2013 | | Nurses' perceptions of sustainability of Magnet® efforts | Magnet® |  |  |  |
| Vega & Da Cunha | | 2023 | | Commensuration of health-care quality standards through hospital accreditation: from measurement weapon to management tool? | EFQM |  |  |  |
| Viswanathan & Salmon | | 2000 | | Accrediting organizations and quality improvement | JCI |  |  |  |
| Wagner | | 2004 | | Is your nursing staff ready for magnet hospital status? An application of the revised Nursing Work Index | Magnet® |  |  |  |
| Wang et al. | | 2015 | | Quality improvements in decreasing medication administration errors made by nursing staff in an academic medical center hospital: a trend analysis during the journey to Joint Commission International accreditation and in the post-accreditation era | JCI | x | x |  |
| Wardhani et al. | | 2019 | | Hospitals accreditation status in Indonesia: associated with hospital characteristics, market competition intensity, and hospital performance? | JCI |  |  |  |
| Warshawsky et al. | | 2015 | | Achieving 80% BSN by 2020: Chief Nurse Executive Role and ANCC Influence | Magnet® | x |  |  |
|  |  |  |  |  | Pathway to Excellence® |  |  |  |
| Weeks et al. | | 2006 | | Certification and magnet hospitals - Will certification advance your career and improve patient outcomes? | Magnet® | x |  |  |
| Weeks et al. | | 2007 | | The ABCs of organizational credentialing | Magnet® |  |  |  |
| Wolf et al. | | 2014 | | The developmental levels in achieving Magnet® designation, part 1 | Magnet® | x |  |  |
| Wonder | | 2013 | | Work engagement in Magnet(®)-designated hospitals: Exploring social and institutional demographics of RNs to optimize improvement efforts | Magnet® |  |  |  |
| Wonder et al. | | 2017 | | Loss of Magnet® Designation and Changes in RN Work Engagement: A Report on How 1 Hospital's Culture Changed Over Time | Magnet® |  |  |  |
| Yildiz et al. | | 2014 | | Perceptions of nurses on the impact of accreditation on quality of care | JCI |  |  |  |
|  |  |  |  |  | Qmentum® |  |  |  |
|  |  |  |  |  | EQuIP |  |  |  |
| Yildiz et al. | | 2019 | | Effect of accreditation and certification on the quality management system: Analysis based on Turkish hospitals | ISO 9001 |  |  |  |
|  |  |  |  |  | JCI |  |  |  |
| **Number of included studies by scheme:** | | |  |  |  |  |  |  |
| **EFQM** | **3** | |  |  |  |  |  |  |
| **EQuIP** | **1** | |  |  |  |  |  |  |
| **ISO 9001** | **12** | |  |  |  |  |  |  |
| **JCI** | **47** | |  |  |  |  |  |  |
| **Magnet** | **77** | |  |  |  |  |  |  |
| **Pathway** | **2** | |  |  |  |  |  |  |
| **Qmentum** | **1** | |  |  |  |  |  |  |
